# Electric field control of magnetization reorientation in Co/Pb (Mg_1/3_Nb_2/3_)-PbTiO_3_ heterostructure

**DOI:** 10.1186/s11671-017-1866-6

**Published:** 2017-02-09

**Authors:** Fenglong Wang, Cai Zhou, Dunzhu Gesang, Changjun Jiang

**Affiliations:** Key Laboratory for Magnetism and Magnetic Materials of MOE, Lanzhou University, Lanzhou, 730000 People’s Republic of China

**Keywords:** Multiferroic heterostructure, Magnetization reorientation, Strain-mediated magnetoelectric coupling

## Abstract

Herein, we demonstrated an apparent electric field control of magnetization reorientation at room temperature, through a strain-mediated magnetoelectric coupling in ferromagnetic/ferroelectric (FM/FE) multiferroic heterostructure. As the applied electric field increased, the magnetization tended to deviate from the original direction, which was induced by nonlinear strain *vs* electric-field behavior from the ferroelectric substrates. Ferromagnetic resonance showed that the in-plane magnetic easy axis of the Co film was shifted sharply with electric field *E* = 10 kV/cm, which indicates that the in-plane uniaxial magnetic anisotropy of the Co film can be inverted via the application of an electric field. These results demonstrated that converse magnetoelectric effect in the FM/FE heterostructure was indeed a feasible method to control magnetization orientation in technologically relevant ferromagnetic thin films at room temperature.

## Background

Controlling of the magnetic anisotropy or magnetization directly by using electric field [1–3] is of great importance not only in fundamental research, but also in technological applications, including high density information storage, ultra-low power-consuming magnetoelectric devices and sensors [4–6]. Recently, room-temperature electric field manipulate magnetism has been achieved by converse magnetoelectric effect (CME) [7, 8] in multiferroic composites such as a heterostructure of ferroelectric (FE) and ferromagnetic (FM) [9, 10]. There have been a number of studies on artificial multiferroic composite heterostructure with different ferroelectric substrates such as [Pb(Mg_1/3_Nb_2/3_)O_3_]_1-x_-[PbTiO_3_]_x_ (PMN-PT) [11, 12], BaTiO_3_ (BTO) [13], PbZr_1-x_Ti_x_O_3_ (PZT) [14], BiFeO_3_ (BFO) [15], and with magnetic materials such as CoFe_2_O_4_, Co, FeGaB, Co_0.9_Fe_0.1_. In such multiferroic composites, several mechanisms have been proposed to control magnetization in multiferroic heterostructure, including exchange couplling [16], exchange bias from ferroelectric-antiferromagnetic substrates, charge accumulation/dissipation at a multiferroic interface [17], and strain transferred from the ferroelectric layer to the ferromagnetic layer [18]. Considering the requirements of applications, large magnetic response to electric stimuli should be performed at room temperature and therefore the strain-mediated magnetoelectric (ME) effect is one of the most promising candidates since strong coupling between ferroelectric and ferromagnetic phases at room temperature can be achieved across their interfaces, leading to a large magnetic response to electric stimuli in such heterostructure. In the present work, we employed single crystal Pb (Mg_1/3_Nb_2/3_) -PbTiO_3_ (PMN-PT) as a substrate and fabricated Co film by sputtering onto PMN-PT substrate with oblique sputtering, which induced in-plane uniaxial anisotropy. We investigated static and dynamic magnetic properties of Co films with applying electric field and demonstrated an obviously electric-field control of magnetization reorientation in Co/PMN–PT heterostructure at room temperature via strain-mediated CME coupling.

## Methods

**Fig. 1:**
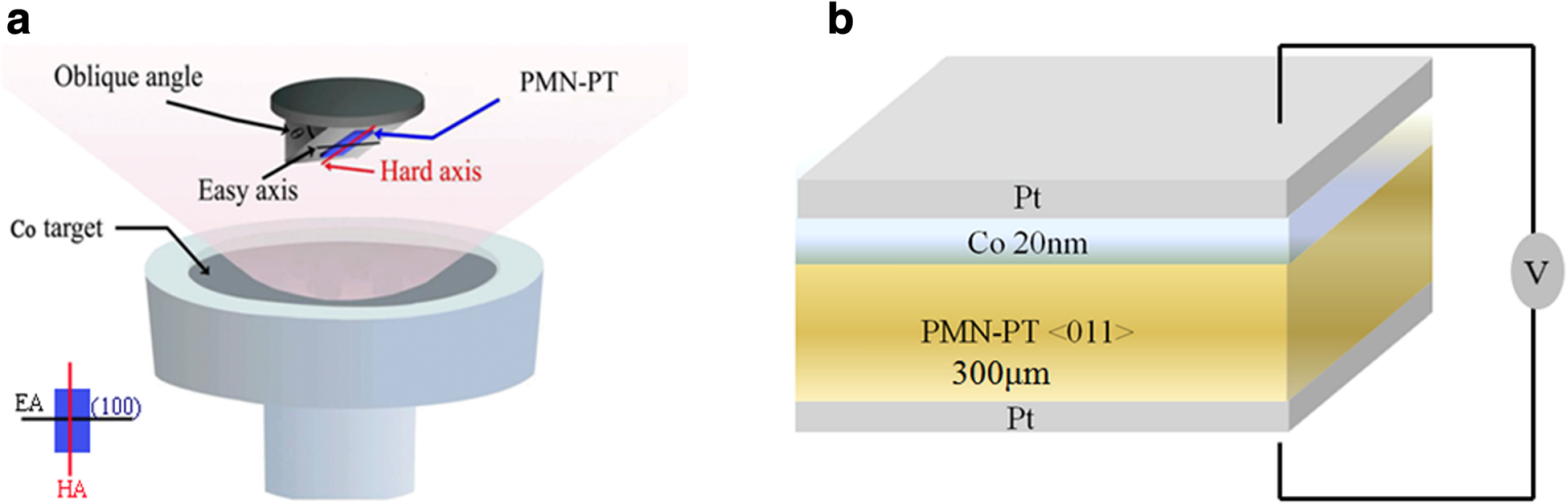
**a** Schematic illustration of the sputtering arrangement. **b** Schematic of layers structure

Co thin films was deposited on a PMN-PT (011) (thickness is 300 μm) substrate by radio frequency magnetron sputtering, as shown in Fig. 1a. The background pressure was lower than 5 × 10^-5^ Pa. For the deposition of Co layers, a Co target, 75 mm in diameter and 3 mm in thickness was used as the sputtering target, and the argon gas was used as the ambient gas. The sputtering chamber was evacuated to a base pressure 0.2 Pa. The RF power was 50w. The thickness of Co layer was 20 nm. While sputtering, an Ar flow rate of 10 SCCM (SCCM denotes cubic centimeter per minute at STP). Sputtering angle of the film was 20° without applied field. Figure 1b shows the schematic drawing of the sputtering arrangement. The film thickness was measured by the setback instrument (Vecco Dektak 8). The static magnetic properties were measured by vibrating sample magnetometer (VSM) and the dynamic magnetic properties were measured by in-plane ferromagnetic resonance (FMR) measurements were performed in a JEOL, JES-FA 300 (X-band at 8.969 GHz) spectrometer. The microwave permeability measurements of the films were performed by a vector network analyzer (PNA E8363B) with micro-strip method.

## Results and discussion

**Fig. 2:**
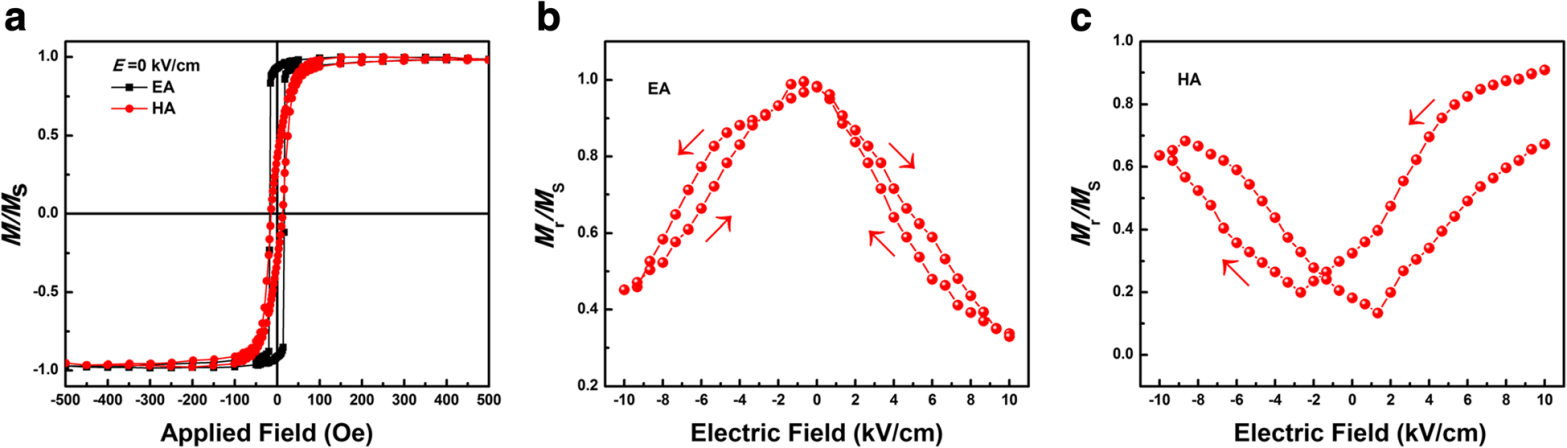
**a** The in-plane hysteresis loops of Co/PMN-PT thin films with *E* = 0 kV/cm. The easy and hard axis remanence of Co/PMN-PT thin films with different *E*: (**b**) EA and (**c**) HA

One 20-nm-thick Co layer with oblique sputtering angle 20°was deposited on the face of 300-um-thick PMN-PT substrate. Figure 2a shows the magnetic hysteresis loops of the Co thin film measured at room temperature, where the magnetic field was applied in the film plane, and the DC bias electric field was applied upon the FE substrate layers along their thickness direction. From Fig. 2a, before the polarization of PMN-PT, the Co film shows an evident in-plane uniaxial magnetic anisotropy. Hysteresis loop of the easy magnetization direction was substantially a rectangle, while remanence ratio (*M*_r_ */M*_s_) was close to 1. Such an obvious in-plane uniaxial magnetic anisotropy is mainly resulted by the geometry of the deposition system, such as oblique incidence [19]. To study the correlation between the change of magnetization and electric field directly, the in-plane remanence alone easy axis (EA) and hard axis (HA) of the Co layer were measured by VSM without magnetic field under different electric field as shown in Fig. 2b, c. Figure 2b, c shows that with the increase of the electric field, the remanence along EA is reduced, while increased the remanence in the hard axis, which indicated that a rotation of magnetic EA would be obtained. The magnetization response to electric field exhibits a symmetrical butterfly-like behavior, similar to previous reports [20] in other FM/FE heterostructure, which attributed to piezostrain effect from FE substrate.

**Fig. 3:**
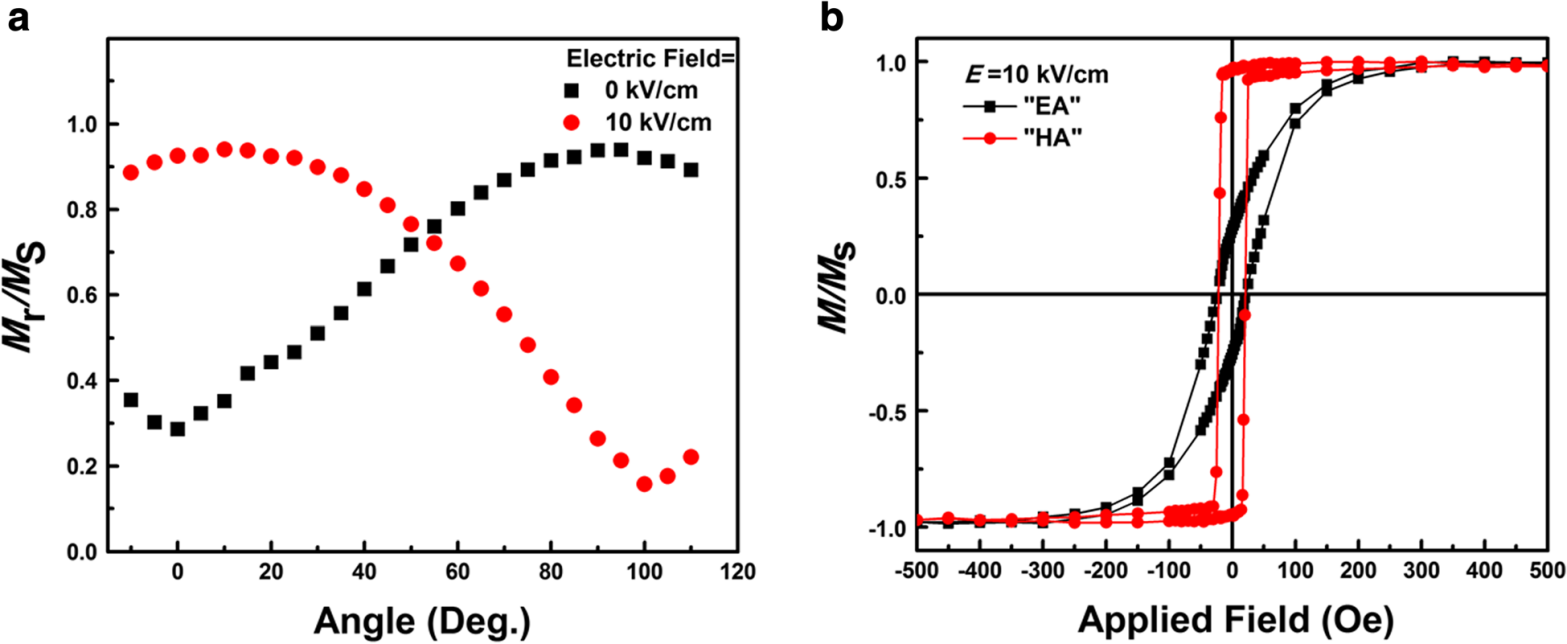
**a** The rotational magnetization curves under different electric field. **b** The in-plane hysteresis loops of Co/PMN-PT thin films with *E* = 10 kV/cm

In order to demonstrate magnetic easy-axis reorientation, the rotational magnetization curve (RMC) was measured, as shown in Fig. 3a. Here, before measuring an H-field with 500 Oe (higher than the saturation field) was applied along each measuring direction. From Fig. 3a, maximum position of the remanence ratio was obtained at 90° (*E* = 0 kV/cm) and 10° (*E* = 10 kV/cm), respectively, which suggested that the magnetic EA rotated obviously. In addition, Fig. 3b showed the magnetic hysteresis loops with *E* = 10 kV/cm in the original EA and HA direction. Obviously, the remanence ratio of original HA was close to 1, which was another evidence of the occurrence of magnetic moment rotation. This giant electrical modulation of magnetization originated from the combination of the ultra-high value of anisotropic in-plane piezoelectric coefficients of (011)-cut PMN-PT and the perfect soft ferromagnetism with in-plane uniaxial anisotropy of Co film. The stress from PMN-PT, which was induced by the electric field, can be transferred to the deposited magnetic Co layer, and formed stress anisotropy. The change of the magnetization resulted from the competition between stress anisotropy and uniaxial anisotropy, which driven the magnetization to deviate from the original position.

**Fig. 4:**
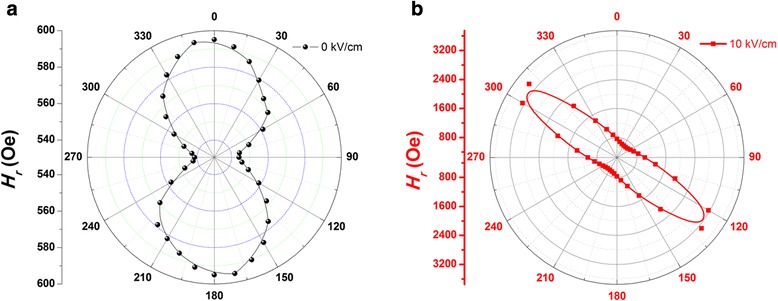
the FMR resonance field (*H*_r)_ obtained for a constant actuator electric field: **a** 0 kV/cm and **b** 10 kV/cm

Generally, magnetic thin film application is based on analysis of the dynamic magnetic or magnetization process, which are subjected to an effective magnetic anisotropy field *H*_eff_ as given by the Landau-Lifshitz-Gilbert (LLG) equation [21]

1 $$ \frac{dM}{dt}=-\gamma \left( M\times {H}_{\mathrm{eff}}\right)+\frac{\alpha}{M_S} M\times \frac{dM}{dt}, $$

where *M*_S_ is representing saturation magnetization, *H*_eff_ is effective magnetic anisotropy field, *γ* is the gyromagnetic factor, *α* is damping constant. Equation (1) illustrated that the dynamic magnetic properties of the magnetic film be able to be regulated through control the size and/or direction of the *H*_eff_. Based on above VSM results, an E-field controlled easy-axis reorientation was obtained, which the direction of the in-plane anisotropy field was adjusted. The dynamic magnetoelectric response of the films was investigated using the more direct and sensitive FMR spectroscopy at room temperatures under different electric field. The external magnetic field was along in-plane direction of the thin film and the electric field were applied upon the FE substrate layers along [011] direction (as shown in Fig. 1b). The relation of resonance field *vs* angles between external magnetic field and the EA direction were obtained, as shown in the Fig. 4. Co/PMN-PT heterostructure thin film showed obviously in-plane uniaxial anisotropy at *E* = 0 kV/cm, which was consistent with the results of VSM in the Fig. 2a. However, the easy axis is rotated obviously as the electric field increased from 0 to 10 kV/cm. Moreover, effective magnetic anisotropy field was also changed from 26 Oe at *E* = 0 kV/cm to 1361 Oe at *E* = 10 kV/cm. Similar to the previous discussion, upon the application of an electric field the PMN-PT substrate would produce in-plane anisotropic stress on the Co film which assisted the inducement of the in-plane magnetic anisotropy. Although there existed a competition between stress-induced magnetic anisotropy and the established magnetic anisotropy induced from oblique deposition, the former was much larger than the latter, so that the total effective magnetic anisotropy was enhanced. Therefore, The FMR results distinctly demonstrated electric field tuning the in-plane magnetic anisotropy in Co/PMN-PT heterostructure.

**Fig. 5:**
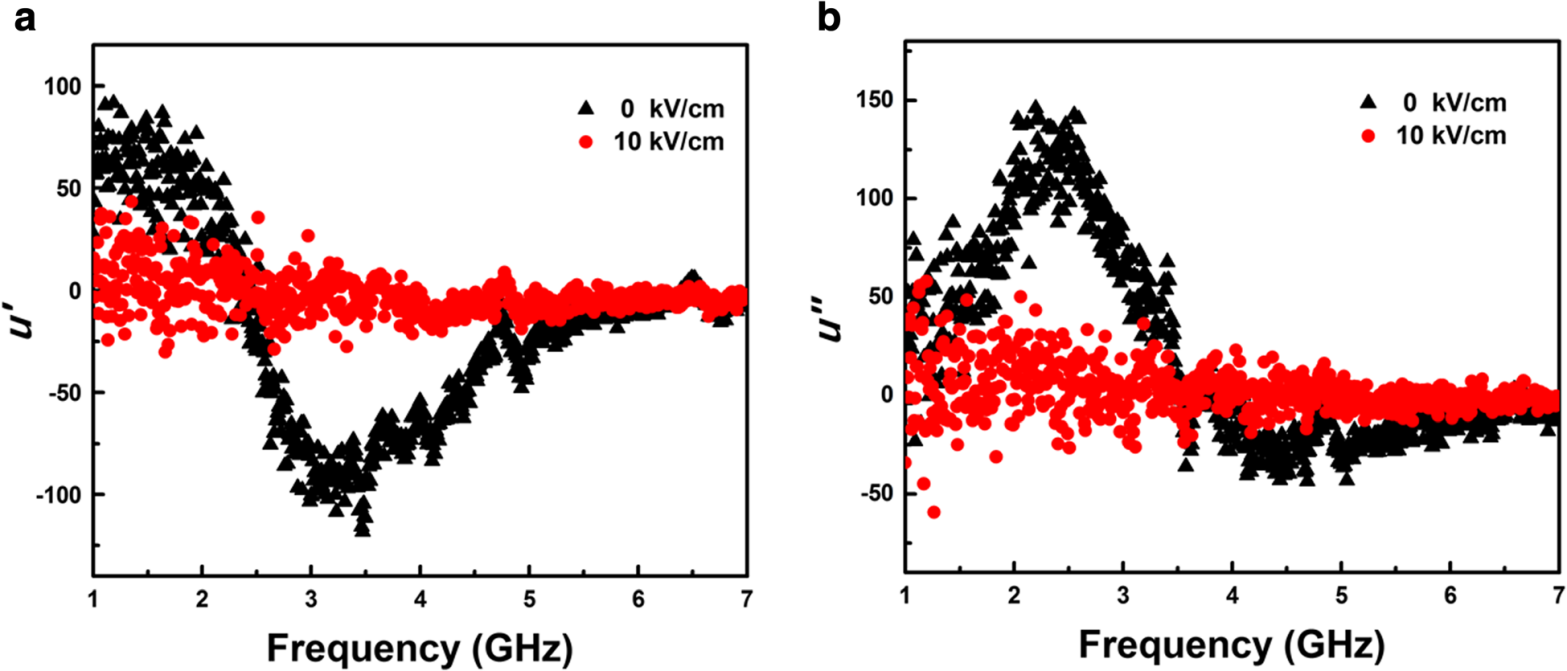
**a** Real *μ'*. **b** Imaginary *μ"* permeability spectra of Co/PMN-PT film under the electric field *E* = 0 and 10 kV/cm

Usually, the influences of magnetic interactions could be much more salient in the high-frequency magnetization dynamics and the magnetization rotation can also be well understood by means of vector network analyzer measurement. Figure 4 shows the dependence of complex permeability *μ = μ' – j μ"* on frequency for the films with different electric field measured by microstrip method using vector network analyzer (PNA E8363B). The *μ'* and *μ"* represent the real and imaginary part of complex permeability, respectively. Figure 5 showed that the resonance frequency was 2.4 GHz with zero electric field. However, the resonance absorption peak was not observed under *E* = 10 kV/cm. This result can be explained by following reason: with *E* = 0 kV/cm, the orientation of the magnetization was perpendicular to the direction of microwave magnetic field, resulted to the magnetization precession, which was detected a resonance peak. While for *E* = 10 kV/cm, according to the results of VSM and FMR, the magnetization deviated from the original position due to applying an electric and was along microwave magnetic field, which resulted to magnetization precession disappear. This finding has a promising potential for the electric controlling of microwave devices, especially phase shift device, anti-electromagnetic interference etc.

## Conclusions

The static and dynamic magnetic properties of Co/PMN-PT films with applying electric field have been investigated. Butterfly-shaped remanence versus electric field loops were obtained for the Co/PMN–PT thin films, which essentially tracks the strain loop in the single PMN–PT film. Ferromagnetic resonance shows that the magnetic easy axis in the Co film plane is rotated dramatically with applying an electric field, which indicated that the in-plane uniaxial magnetic anisotropy of the Co film can be inverted via the application of an electric field. The competition between the as-deposited magnetic anisotropy and stress anisotropy from FE substrate resulted to reorientation of magnetization. In particularly, electric field tuning the disappearance of resonance peak has crucial application in microwave devices, such as microwave absorption, etc.

## Abbreviations

BFO: BiFeO_3_
BTO: BaTiO_3_
CME: Converse magnetoelectric effect
EA: Easy axis
FM/FE: Ferromagnetic/ferroelectric
HA: Hard axis
ME: Magnetoelectric
PMN-PT: [Pb(Mg_1/3_Nb_2/3_)O_3_]_1-x_-[PbTiO_3_]_x_
PZT: PbZr_1-x_Ti_x_O_3_
RMC: Rotational magnetization curve
